# 16S rRNA gene sequencing reveals altered gut microbiota in young adults with schizophrenia and prominent negative symptoms

**DOI:** 10.1002/brb3.3579

**Published:** 2024-06-06

**Authors:** Yi‐Huan Chen, Huan Yu, Fen Xue, Jie Bai, Li Guo, Zheng‐Wu Peng

**Affiliations:** ^1^ Department of Psychiatry Xijing Hospital Air Force Medical University Xi'an China; ^2^ Department of Psychiatry Gaoxin Hospital Xi'an China

**Keywords:** gut microbiota, negative symptoms, schizophrenia, young adults

## Abstract

**Background:**

Gut dysbiosis has been established as a characteristic of schizophrenia (SCH). However, the signatures regarding SCH patients with prominent negative symptoms (SCH‐N) in young adults have been poorly elucidated.

**Methods:**

Stool samples were obtained from 30 young adults with SCH‐N, 32 SCH patients with prominent positive symptoms (SCH‐P) along with 36 healthy controls (HCs). Microbial diversity and composition were analyzed by 16S rRNA gene sequencing. Meanwhile, psychiatric symptoms were assessed by the positive and negative syndrome scale (PANSS).

**Results:**

There is a significant difference in β‐diversity but not α‐diversity indexes among the three groups. Moreover, we found a higher abundance of *Fusobacteria* and *Proteobacteria* phyla and a lower abundance of *Firmicutes* phyla in SCH‐N when compared with HC. Besides, we identified a diagnostic potential panel comprising six genera (*Coprococcus*, *Monoglobus*, *Prevotellaceae_NK3B31_group*, *Escherichia‐Shigella*, *Dorea*, and *Butyricicoccus*) that can distinguish SCH‐N from HC (area under the curve = 0.939). However, the difference in microbial composition between the SCH‐N and SCH‐P is much less than that between SCH‐N and the HC, and SCH‐N and SCH‐P cannot be effectively distinguished by gut microbiota.

**Conclusion:**

The composition of gut microbiota was changed in the patients with SCH‐N, which may help in further understanding of pathogenesis in young adults with SCH‐N.

## Graphical abstract

Gut microbiome is altered in young adult patients with SCH‐N six genera that can distinguish SCH‐N from HC were identified.

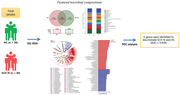

## 1 INTRODUCTION

Schizophrenia (SCH) is considered one of the most severe psychiatric disorders (Jauhar et al., 2022), with low remission rates, high mortality rates, and a high global disease burden (Hansen et al., 2022). It typically develops in young adulthood; men tend to have an onset that peaked around the age of 22, while women showed a more plateau‐like phase, which started after the age of 20 and only slightly decreased over the years up until the age of 65 (Sommer et al., 2020){Sommer, 2020 #1}. Similarly, the lifetime prevalence of SCH was 0.6%, and the highest prevalence of SCH is 1.3% among those aged 18–34 years in China (Y. Huang et al., 2019). This pattern of high incidence in young adults makes SCH have more negative effects on social cognition and psychological development (Hollis, 2000). Although antipsychotics play an integral role in the treatment of young adult patients with SCH, the adverse effects such as rapid weight gain (Alvarez‐Jimenez et al., 2008), disturbances in bone growth and sexual development cannot be ignored (Calarge et al., 2013; Lally & MacCabe, 2015). Moreover, young adults are more likely to exhibit negative symptoms but not delusions and hallucinations than adults (Strauss et al., 2020).

Negative symptoms are core components of SCH, which include avolition, blunted affect, asociality, alogia, and anhedonia. Negative symptoms account for a large proportion of poor functional outcomes and long‐term disability in areas such as impaired social functioning, academic and occupational performance, and quality of life (Correll & Schooler, 2020). It can also be secondary symptoms that are related to positive symptoms, adverse effects of treatment, or comorbid depression (Begue et al., 2020). Generally, negative symptoms showed less response to antipsychotics and may even be exacerbated in some cases (McCutcheon et al., 2020), up to 90% of SCH patients with a first psychotic episode have at least one negative symptom, and 35%–70% of patients still have negative symptoms after treatment (An der Heiden et al., 2016; Makinen et al., 2008). It is necessary to explore its pathogenesis from a perspective other than regulating neurotransmitters and develop new therapeutic strategies.

The correlation between intestinal flora imbalance in mental diseases has been widely reported. Indeed, multiple studies elucidate dysbiosis of the intestinal flora in patients with SCH (Szeligowski et al., 2020; Yuan et al., 2019; P. Zheng et al., 2019). Notably, negative symptoms can be influenced by microbes through the microbiota‐gut‐brain axis (C. Zhu et al., 2021). A recent review focused on the relationship between gut microbiota and clinical symptoms of SCH, mentioning changes in the gut microbiome associated with the severity of negative symptoms (Nocera & Nasrallah, 2022), and a systematic review also suggested that probiotics may have some positive effects on SCH symptoms (Ng et al., 2019). However, to date, few studies have directly investigated the difference in the compositions of gut microbiota between healthy control (HC) and SCH with prominent negative symptoms (SCH‐N), as well as the difference between SCH‐N and SCH with prominent positive symptoms (SCH‐P) in young adults. Exploring the microbial characteristics and potential mechanisms may further clarify potential intervention targets for SCH‐N in young adults.

Together, the objectives of this study are to compare the differences in gut microbiota composition between HC and SCH‐N, as well as SCH‐N and SCH‐P in young adults, identify the potential gut microbial biomarkers of SCH‐N, and investigate the association between the clinical variables and gut microbiota composition.

## 2 METHODS

### 2.1 Participant selection

The study was registered with the Chinese Clinical Trial Registry (No. ChiCTR‐ROC‐17013029) and was approved by the Clinical Research Ethics Committee of Xijing Hospital (Approve No. KY20172048‐F‐2, October 19, 2017). The protocol was performed in accordance with the Helsinki Declaration, and all subjects signed written informed consent and volunteered to participate.

All the patients met the diagnostic of SCH‐N according to the Statistical Clinical Interview for DSM‐5 by two psychiatrists. Meanwhile, patients with a total positive and negative syndrome scale (PANSS) score ≥60, at least three out of seven items of PANSS negative subscale score ≥4, or at least two items score ≥5, and a total PANSS negative subscale score ≥20, and at least 3 points more than the total PANSS positive subscale score were required (Rabinowitz et al., 2013). Patients met the diagnostic of SCH‐P with a total PANSS score ≥60, at least three out of seven items of PANSS positive subscale score ≥4, or at least two items score ≥5, and a total PANSS positive subscale score ≥20, and at least 3 points more than the total PANSS negative subscale score were required. Healthy subjects (HC) were screened through a semi‐structured clinical interview to exclude those with mental illness and taking the participants’ past medical history and combining it with their physical examination results to exclude those with physical illness. All subjects were between the ages of 18 and 30 years and had not taken antibiotics, probiotics, prebiotics, and probiotic‐fermented foods (such as yogurt) within 1 month before enrollment. At the same time, we implemented the following exclusion criteria: (1) digestive diseases; (2) obesity, defined as body‐mass index (BMI) ≥28.0; hyperlipidemia, defined as triglyceride levels ≥2.3 mmol/L; hypertension, systolic blood pressure ≥140 mmHg or diastolic blood pressure ≥90 mmHg; hyperglycemia, defined as fasting blood glucose (FBG) ≥6.1 mmol/L; and/or someone who has been diagnosed and/or treated with diabetes; (3) severe dietary imbalance subjects, such as those with high‐fat diet preference or long‐term vegetarians; (4) lactating or pregnant women; (5) subjects with other psychiatric disorders that meet the DSM‐5 diagnostic criteria; (6) taken a full dose of any type of psychotropic medication for more than two consecutive days in the 2 weeks before the study begins.

Thirty‐six SCH‐N and 36 SCH‐P patients were recruited from the Department of Psychiatry, and 43 healthy individuals were recruited from the health screening center of the hospital as controls. According to the inclusion and exclusion criteria, 17 subjects were excluded. The reasons were as follows: one subject had elevated blood glucose, blood pressure, and lipid levels, two subjects had elevated lipid levels and blood glucose, three subjects had elevated blood pressure only, one subject had elevated blood glucose and subclinical hypothyroidism, four subjects had elevated lipid levels only, two subjects had a BMI over 28, one subject had used antibiotics in the last month, and three subjects had consumed yogurt containing probiotics in the last 3 days.

### 2.2 Fecal sample collection and 16S rRNA microbiome sequencing

Fresh fecal samples were quickly collected in a sterile cryotube after defecation and quick‐frozen in liquid nitrogen immediately before analyses. The E.Z.N.A. Stool DNA Kit (Omega Bio‐Tek) was used to extract genomic DNA from the samples (Yan et al., 2020). The V3–V4 hypervariable regions of bacterial 16S rRNA gene were amplified by polymerase chain reaction using primers 338F (5′‐ACTCCTACGGGAGGCAGCAG‐3′) and 806R (5′‐GGACTACHVGGGTWTCTAAT‐3′). Amplicons were pooled in equimolar and paired‐end sequenced (2 × 250 bp) on an Illumina MiSeq PE300 platform after being extracted and quantified. USEARCH 8.0, UPARSE (version 7.1; http://drive5.com/uparse/), and ribosomal database project classifier algorithm (http://rdp.cme.msu.edu/) were used to perform Raw FASTQ files analysis, sequences assigned to operational taxonomic units (OTUs), and taxonomy analysis, respectively. In detail, samples were distinguished according to the barcode and primers, and the sequence direction was adjusted, exact barcode matching, and two nucleotide mismatches in primer matching. Then the optimized sequences were clustered into OTUs using Usearch ll with a 97% sequence similarity level. The most abundant sequence for each OTU was selected as a representative sequence. The taxonomy of each OTU representative sequence was analyzed by RDP Classifier version 2.13 against the l6S rRNA gene database using a confidence threshold of 0.7. Then, the QIIME software (version 1.9.1), R project Vegan package (version 2.5.3), R project ggplot2 package (version 2.2.1), and R project pheatmap package (version 3.3.1) were used to analyze the subsequent, α‐diversity, principal coordinate analysis (PCoA) linear discriminant analysis (LDA) and correlation analysis on the cloud platform of Majorbio Bio‐Pharm Technology Co., Ltd.

### 2.3 Statistical analysis

SPSS version 19.0 and GraphPad Prism 9.0 were used for analyzing data and plotting. Comparisons of sociodemographics, clinical variables, and α‐diversities differences among the three groups were performed using the *χ* ^2^ test for categorical variables, one‐way analysis of variance was used for variables that satisfied variance *χ* ^2^ and normal distribution, and Bonferroni was applied for post hoc tests. Nonparametric tests were used for variables that did not satisfy *χ* ^2^ and normal distribution. The Shapiro–Wilk test was applied to detect normal distribution. Bioinformatic analysis of the gut microbiota was performed using the Majorbio Cloud platform (https://cloud.majorbio.com). Venn analysis between groups was performed in the R project VennDiagram package (version 1.6.16). The community bar diagram visualizes the composition of the dominant flora at the genus level in each group. The similarity among the microbial communities in different samples was determined by PCoA based on unweighted unifrac, weighted unifrac, and Bray‐Curtis dissimilarity using the Vegan package (version 2.5.3). The PERMANOVA test was used to assess the percentage of variation explained by the treatment along with its statistical significance. The linear discriminant analysis effect size (LEfSe) (http://huttenhower.sph.harvard.edu/LEfSe) was performed to identify the significantly abundant taxa of bacteria. Spearman's rank correlation coefficient between clinical variables and genera was calculated in the R project heatmap package (version 3.3.1). The R random forest package was used to build classification models using profiles of genera, followed by ten‐fold cross‐validation. SPSS version 19.0 was used for receiver operating characteristic (ROC) analysis, and then GraphPad Prism 9.0 was used to draw ROC curves. The ratio of training and testing was 7:3, and the point with the lowest error rate was selected. The *p*‐values were set as two‐tailed with the significance level *α* = .05.

## 3 RESULTS

### 3.1 Sociodemographics and clinical variables of the recruited subjects

A total of 30 SCH‐N patients, 32 SCH‐P patients, and 36 HCs were included. There is no significant difference in terms of sex ratio (*p* = .071), age (*p* = .135), smoking (*p* = .322), alcohol intake (*p* = .382), BMI (*p* = .175), or FBG (*p* = .075), which were found between the SCH‐N and HC groups (Table 1). The total PANSS scores, positive subscales of PANSS scores, and negative subscales of PANSS scores in the SCH‐N group were higher, whereas the global assessment function (GAF) scores in the SCH‐N group were lower than that of the HC group (all *p* < .01). There is no significant difference in terms of sex ratio (*p* = .116), age (*p* = .790), smoking (*p* = .878), alcohol intake (*p* = .467), family history (*p* = .861), or BMI (*p* = .835) between the SCH‐N and SCH‐P groups (Table 2). FBG, the total PANSS scores, and positive subscales of PANSS scores in the SCH‐P group were higher than that of the SCH‐N group (all *p* < .001). The total disease duration, GAF scores, and negative subscales of PANSS scores in the SCH‐N group were higher than that of the SCH‐P group (all *p* < .01). The detailed clinical characteristics of each individual are displayed in Table S1.

**TABLE 1:** Comparisons of sociodemographics, clinical variables and α‐diversity estimations between the healthy control (HC) and schizophrenia with prominent negative symptoms (SCH‐N).

Variable	HC (*n* = 36)	SCH‐N (*n* = 30)	*χ* ^2^ / *t* / *Z*	*p* value
**Sociodemographic**				
Male, number (%)^a^	16 (44.44%)	20 (66.67%)	*χ* ^2 ^= 3.259	.071
Age, years, (x ± s)^b^	24.67 ± 3.21	23.40 ± 3.59	*t* = 1.514	.135
Smoking, number (%)^a^	5 (13.89%)	7 (23.33%)	*χ* ^2 ^= 0.981	.322
Alcohol intake, number (%)^a^	4 (11.11%)	5 (16.67%)	*χ* ^2 ^= 0.721	.382
Family history, number (%)	–	10 (33.33%)	–	–
Duration of illness, months (M (*P*_25_, *P*_75_))	–	28.00 (11.50, 55.50)	–	–
BMI, kg/m^2^, (x ± s)^b^	22.31 ± 2.90	21.30 ± 3.04	*t* = 1.372	.175
Fasting blood glucose, mmol/L (M (*P*_25_, *P*_75_))^c^	4.65 (4.42,4.92)	4.46 (4.38, 4.64)	*Z* = −1.778	.075
PANSS total score, x ± s^b^	40.58 ± 6.03	79.17 ± 8.07	*t* = −22.202	<.001
PANSS negative score, M (*P*_25_, *P*_75_)^b^	7.00 (7.00, 7.00)	24.00 (22.00, 27.00)	*Z* = −7.351	<.001
PANSS positive score, M (*P*_25_, *P*_75_)	7.00 (7.00, 7.00)	16.00 (11.00, 18.00)	*Z* = −6.709	<.001
GAF score, M (*P*_25_, *P*_75_)	96.50 (92.00, 99.00)	37.00 (34.00, 42.00)	*Z* = −3.466	.001
**α‐diversity estimations and observed OTUs**				
Ace, M (*P*_25_, *P*_75_)^c^	354.26 (329.06, 391.80)	387.82 (312.25, 444.57)	Z = −0.721	.471
Chao 1 (x ± s)^c^	365.97 ± 58.13	364.70 ± 83.40	Z = −0.219	.827
Shannon, M (*P*_25_, *P*_75_)^c^	3.61 (3.41, 3.86)	3.43 (3.03, 3.96)	Z = −1.030	.303
Simpson, M (*P*_25_, *P*_75_)^c^	0.06 (0.05, 0.08)	0.08 (0.04, 0.14)	Z = −0.670	.503
Observed‐species, M (*P*_25_, *P*_75_)^c^	289.00 (265.50, 329.50)	286.50 (217.50, 347.25)	Z = −0.786	.432

Abbreviations: BMI, body‐mass index; GAF, global assessment function; OTUs, operational taxonomic units; PANSS, positive and negative syndrome scale.

^a^ *χ* ^2^ analysis.

^b^ Student's *t*‐test.

^c^ Mann–Whitney U test.

**TABLE 2:** Comparisons of sociodemographics, clinical variables, and α‐diversity estimations between the schizophrenia with prominent negative symptoms (SCH‐N) and schizophrenia with prominent positive symptoms (SCH‐P).

Variable	SCH‐N (*n* = 30)	SCH‐P (*n* = 32)	*χ* ^2^ / *t* / *Z*	*p* value
**Sociodemographic**				
Male, number (%)^a^	20 (66.67%)	15 (46.88%)	*χ* ^2 ^= 2.467	.116
Age, years, (x ± s)^b^	23.40 ± 3.59	23.63 ± 3.02	*t* = −0.268	.790
Smoking, number (%)^a^	7 (23.33%)	8 (25.00%)	*χ* ^2 ^= 0.023	.878
Alcohol intake, number (%)^c^	5 (16.67%)	3 (9.38%)	–	.467
Family history, number (%)^a^	10 (33.33%)	10 (31.25%)	*χ* ^2 ^= 0.031	.861
Duration of illness, months (M (*P*_25_, *P*_75_))^d^	28.00 (11.50, 55.50)	12.00 (2.00, 34.00)	*Z* = −2.679	.007
BMI, kg/m^2^, (x ± s)^b^	21.30 ± 3.04	21.16 ± 2.53	*t* = 0.209	.835
Fasting blood glucose, mmol/L (M (*P*_25_, *P*_75_))^d^	4.46 (4.38, 4.64)	4.66 (4.46, 4.95)	*Z* = −2.403	.016
PANSS total score, (x ± s)^b^	79.17 ± 8.07	86.31 ± 8.82	*t* = −3.321	.002
PANSS negative score, M (*P*_25_, *P*_75_)^d^	24.00 (22.00, 27.00)	18.50 (16.00, 20.00)	*Z* = −6.534	<.001
PANSS positive score, M (*P*_25_, *P*_75_)^d^	16.00 (11.00, 18.00)	25.00 (24.00, 28.50)	*Z* = −6.709	<.001
GAF score, M (*P*_25_, *P*_75_)^d^	37.00 (34.00, 42.00)	32.00 (25.25, 35.75)	*Z* = −3.466	.001
**α‐diversity estimations and observed OTUs**				
Ace (x ± s)^b^	364.99 ± 89.16	342.78 ± 77.25	*t* = 1.050	.298
Chao 1 (x ± s)^d^	364.70 ± 83.40	339.92 ± 81.54	*Z* = −0.648	.517
Shannon, M (*P*_25_, *P*_75_)^d^	3.43 (3.03, 3.96)	3.50 (3.12, 3.88)	*Z* = −0.085	.933
Simpson, M (*P*_25_, *P*_75_)^d^	0.08 (0.04, 0.14)	0.08 (0.04, 0.13)	*Z* = −0.014	.989
Observed‐species (x ± s)^d^	278.60 ± 67.54	266.44 ± 69.91	*Z* = −0.796	.426

Abbreviations: BMI, body‐mass index; GAF, global assessment function; OTUs, operational taxonomic units; PANSS, positive and negative syndrome scale.

^a^ *χ* ^2^ analysis.

^b^ Student's *t*‐test.

^c^ Fisher's exact test.

^d^ Mann–Whitney U test.

### 3.2 Sequencing characteristics and gut microbial diversity changes

**FIGURE 1:**
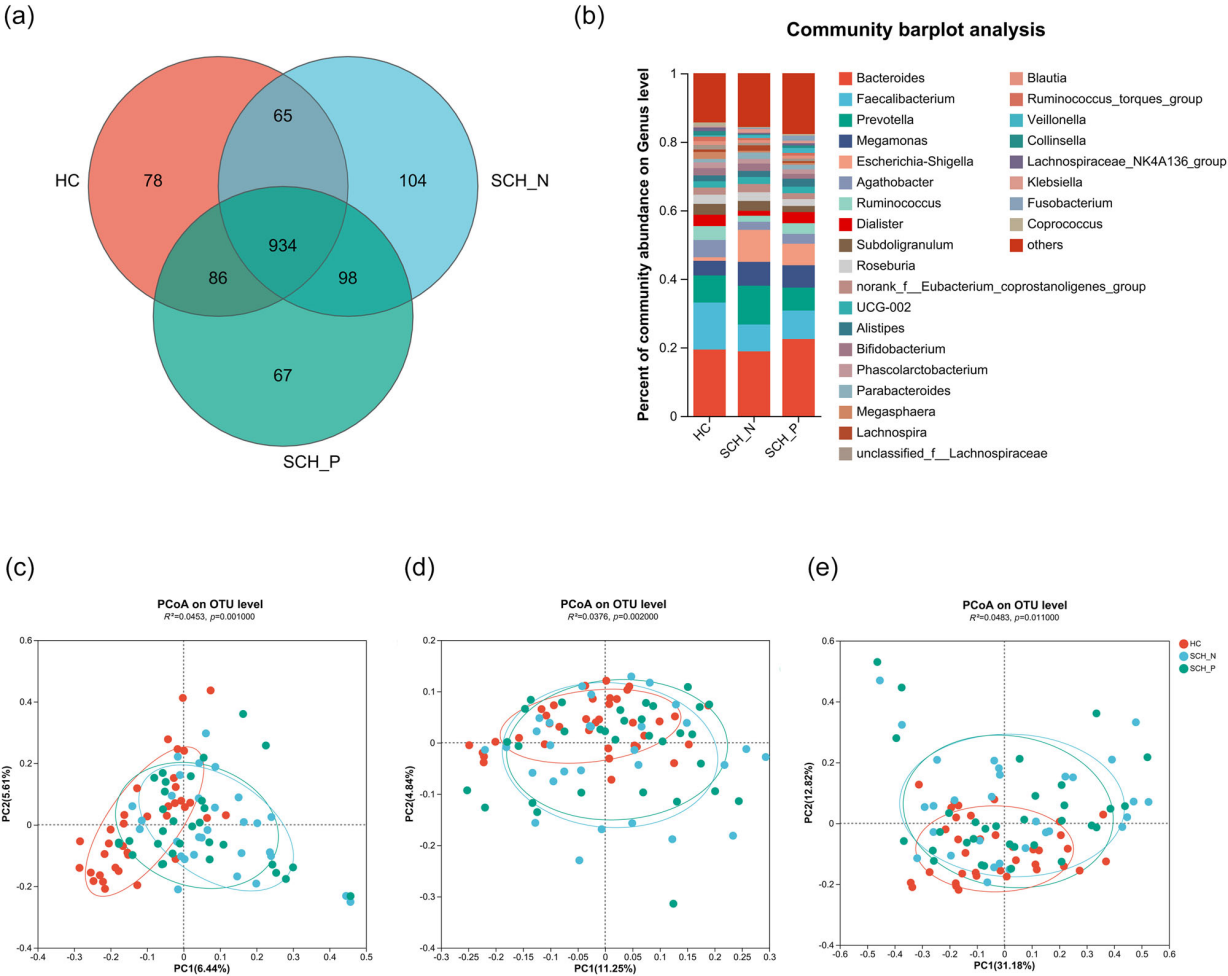
Comparison of species composition and β diversity analysis among healthy controls (HCs), schizophrenia with prominent negative symptoms (SCH‐N) group, and schizophrenia with prominent positive symptoms (SCH‐P) group. (a) The Venn diagram shows the number of common and unique OUTs among the HC, SCH‐N, and SCH‐P groups. (b) The community bar diagram visualizes the percentage of the dominant genera in each group. A clear separation between the three groups by principal co‐ordinate analysis based on Bray–Curtis (c), unweighted UniFrac (d), and weighted UniFrac (e) distance index (all *p* < .05). PCoA, principal coordinate analysis.

We obtained 1,581,511,720 bases and −3,376,112 high‐quality sequences from the stool sample, with an average length of 468.47. Among them, 586,833,799 bases and 1,259,051 high‐quality sequences were obtained from the HC group, 488,832,663 bases and 1,039,261 high‐quality sequences were obtained from the SCH‐N group, whereas 505,845,258 bases and 1,077,800 high‐quality sequences were obtained from the SCH‐P group. Then, 1432 qualified OTUs were clustered at 97% sequence similarity for subsequent analysis (Figure 1a). By community composition analysis, we found the abundance of *Agathobacter* and *Faecalibacterium* was enriched in HC subjects, the abundance of *Escherichia‐Shigella* and *Megamonas* was enriched in SCH‐N patients, and the abundance of *Bacteroides* and *Veillonella* was enriched in SCH‐P patients (Figure 1b).

Different α‐diversity indexes (Chao 1, observed‐species, ACE, Simpson, and Shannon) were used to assess the richness and evenness. In this study, there is no significant difference between HC and SCH‐N in these indices (all *p* > .05) (Table 1). Meanwhile, there is also no significant difference between SCH‐N and SCH‐P in these indices (all *p* > .05) (Table 2). β‐diversity is an indicator of comprehensive microbial phenotypes. In this study, the gut microbiomes of these three groups could be divided into clusters according to microbial‐community composition by Bray Curtis (*R* ^2^ = 0.045, *p* = .001), unweighted UniFrac (*R* ^2^ = 0.038, *p* = .002), and weighted UniFrac (*R* ^2^ = 0.048, *p* = .011), suggesting the gut microbiomes could be clearly separated by PCoA (Figure 1c–e).

### 3.3 Distinct gut microbiome signatures in HC and SCH‐N and their interrelationships with clinical factors

**FIGURE 2:**
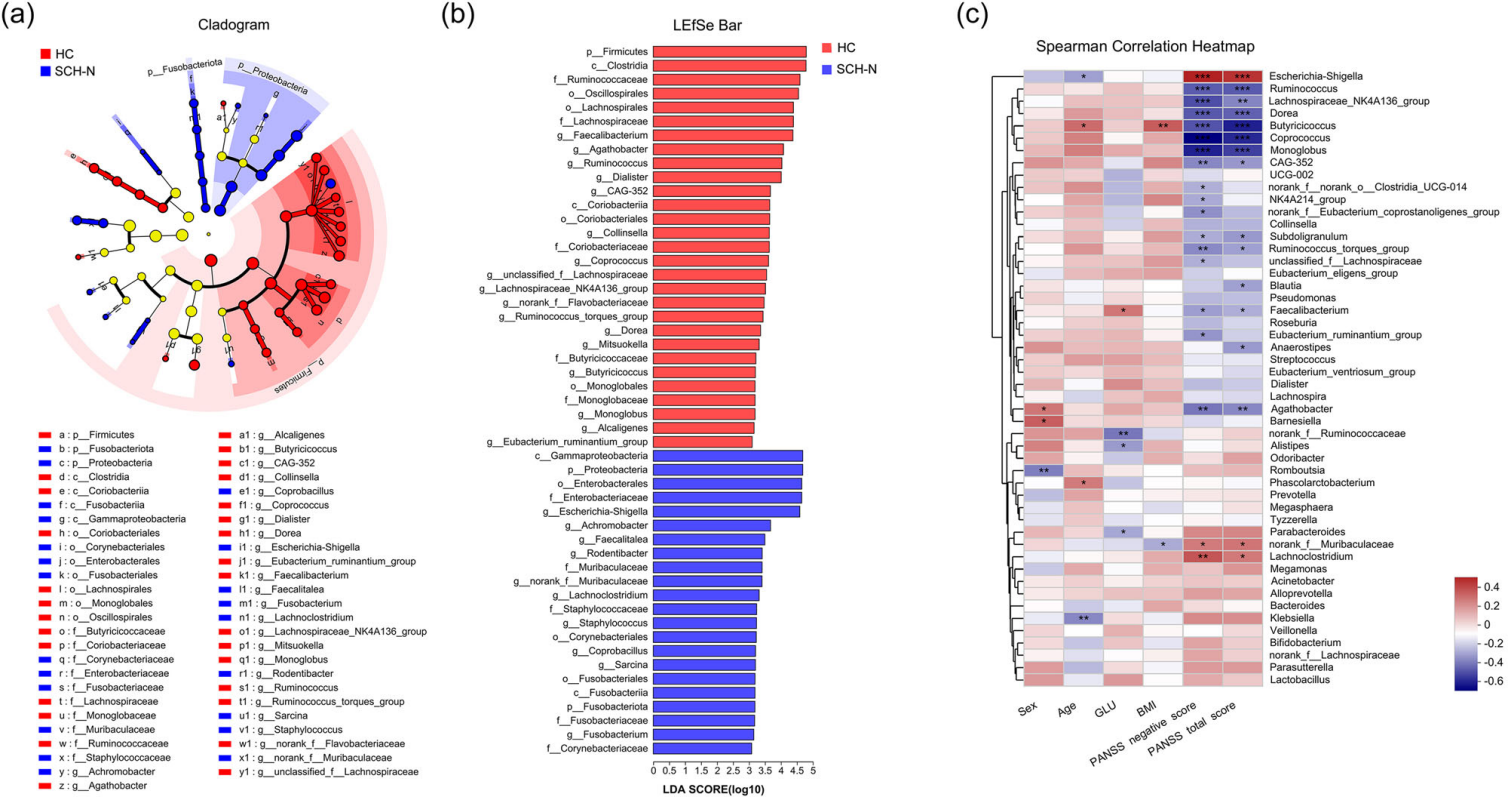
Differences in gut microbiome between healthy control (HC) and schizophrenia with prominent negative symptoms (SCH‐N) groups based on linear discriminant analysis effect size (LEfSe) analysis, and the correlation between gut‐microbiome composition and the clinical parameters. (a) The taxonomic cladogram shows the bacterial taxa enriched in SCH‐N patients (blue dots) and HC (red dots). Yellow dots represent microbial taxa that did not significantly affect the differences between each group. (b) The linear discriminant analysis (LDA) discriminant bar chart shows the microbial taxa with significant differences in the HC (red) and SCH‐N (blue) groups. Larger LDA scores represent a greater effect of species abundance on the different effects. Only taxa with an LDA score >3.0 are shown in the figure. (c) The Spearman correlation heatmap shows the correlation between gut‐microbiome composition and clinical variables. BMI, body‐mass index; GLU, blood glucose; PANSS, positive and negative syndrome scale. *p < .05; **p < .01; ***p < .001.

As shown in Figure 2a,b, at the phylum level, *Fusobacteria* and *Proteobacteria* were enriched in the SCH‐N group, while *Firmicutes* were enriched in the HC group. At the class level, the relative proportions of *Gammaproteobacteria* and *Fusobacteriia* were significantly higher in the SCH‐N group, while *Coriobacteriia* and *Clostridia* were higher in the HC group. At the order level, *Enterobacterales, Corynebacteriales*, and *Fusobacteriales* showed greater enrichment in patients with SCH‐N, whereas *Oscillospirales*, *Lachnospirales*, *Coriobacteriales*, and *Monoglobales* were more abundant in HC. At the family level, there was a higher abundance of *Enterobacteriaceae*, *Muribaculaceae*, *Staphylococcaceae*, *Fusobacteriaceae*, and *Corynebacteriaceae* in the SCH‐N group, while *Ruminococcaceae*, *Lachnospiraceae, Coriobacteriaceae, Butyricicoccaceae*, *and Monoglobaceae* were enriched in the HC group. In addition, we identified 27 genera with different abundances between the HC and SCH‐N groups.

We performed Spearman correlation analysis to investigate the association between the clinical variables and gut‐microbiome composition at the genus level (Figure 2c). *Agathobacter* and *Bamesiella* were enriched in females, whereas *Romboutsia* was enriched in male adults. The abundance of *Butyricicoccus* and *Phascolarctobacterium* was positively correlated, whereas *Escherichia‐Shigella* and *Klebsiella* were negatively correlated with age. *Faecalibacterium* was positively correlated, whereas *norank_f_Ruminococcaceae*, *Alistipes*, and *Parabacteroides* were negatively correlated with FBG. Moreover, the abundance of *Butyricicoccus* was positively correlated, whereas *norank_f_Muribaculaceae* was negatively correlated with BMI. Finally, *Ruminococcus*, *Lachnospiraceae_NK4A136_group*, *Dorea*, *Butyricicoccus*, *Monoglobus*, *CAG‐352*, *Subdoligranulum*, *Ruminococcus_torques_group*, *Faecalibacterium*, and *Agathobacter* were negatively correlated, whereas *Escherichia‐Shigella*, *norank_f_Muribaculaceae*, and *Lachnoclostridium* were positively correlated with the total PANSS scores and negative subscales of PANSS scores.

### 3.4 Distinct gut microbiome signatures in the SCH‐N and SCH‐P groups, and their interrelationships with clinical factors

**FIGURE 3:**
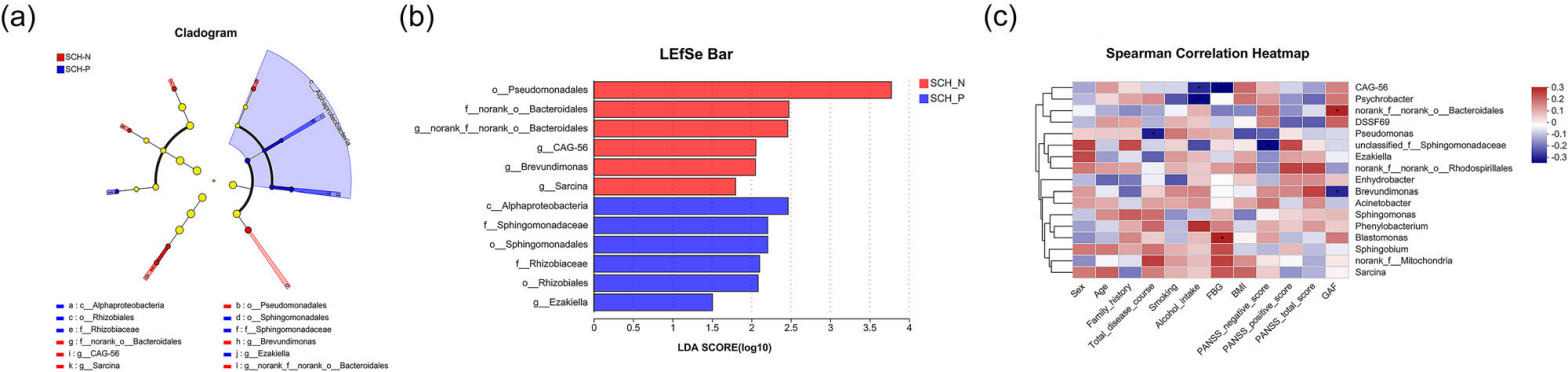
Differences in gut microbiome between schizophrenia with prominent negative symptoms (SCH‐N) and schizophrenia with prominent positive symptoms (SCH‐P) groups based on linear discriminant analysis effect size (LEfSe) analysis, and the correlation between gut‐microbiome composition and the clinical parameters. (a) The taxonomic cladogram shows the bacterial taxa enriched in SCH‐N (red dots) and SCH‐P (blue dots) patients. Yellow dots represent microbial taxa that did not significantly affect the differences between each group. (b) The linear discriminant analysis (LDA) discriminant bar chart shows the microbial taxa with significant differences in the SCH‐N (red) and SCH‐P (blue) groups. Larger LDA scores represent a greater effect of species abundance on the different effects. Only taxa with an LDA score >1.5 are shown in the figure. (c) The Spearman correlation heatmap shows the correlation between gut‐microbiome composition and clinical variables. *p < .05.

We also used LEfSe to find gut flora enriched in the SCH‐N and SCH‐P groups (Figure 3a,b). At the class level, *Alphaproteobacteria* was greatly enriched in the SCH‐P group. At the order level, the relative proportions of *Pseudomonadales* were significantly higher in the SCH‐N group, while *Sphingomonadales* and *Rhizobiales* were more abundant in the SCH‐P group. At the family level, *norank_o_Bacteroidales* showed greater enrichment in patients with SCH‐N, whereas *Sphingomonadaceae* and *Rhizobiaceae* were more abundant in SCH‐P. At the genus level, there was a higher abundance of *norank_f_norank_o_Bacteroidales*, *CAG‐56*, *Brevundimonas*, and *Sarcina* in the SCH‐N group, while *Ezakiella* was enriched in the SCH‐P group. The abundance of *Blastomonas* was positively correlated, whereas *CAG‐56* was negatively correlated with FBG. *CAG‐56* and *Psychrobacter* were negatively correlated with alcohol intake. *Norank_f_norank_o_Bacteroidales* was positively correlated, whereas *Brevundimonas* was negatively correlated with GAF scores (Figure 3c).

### 3.5 Gut microbial biomarkers for discriminating SCH‐N from HC

**FIGURE 4:**
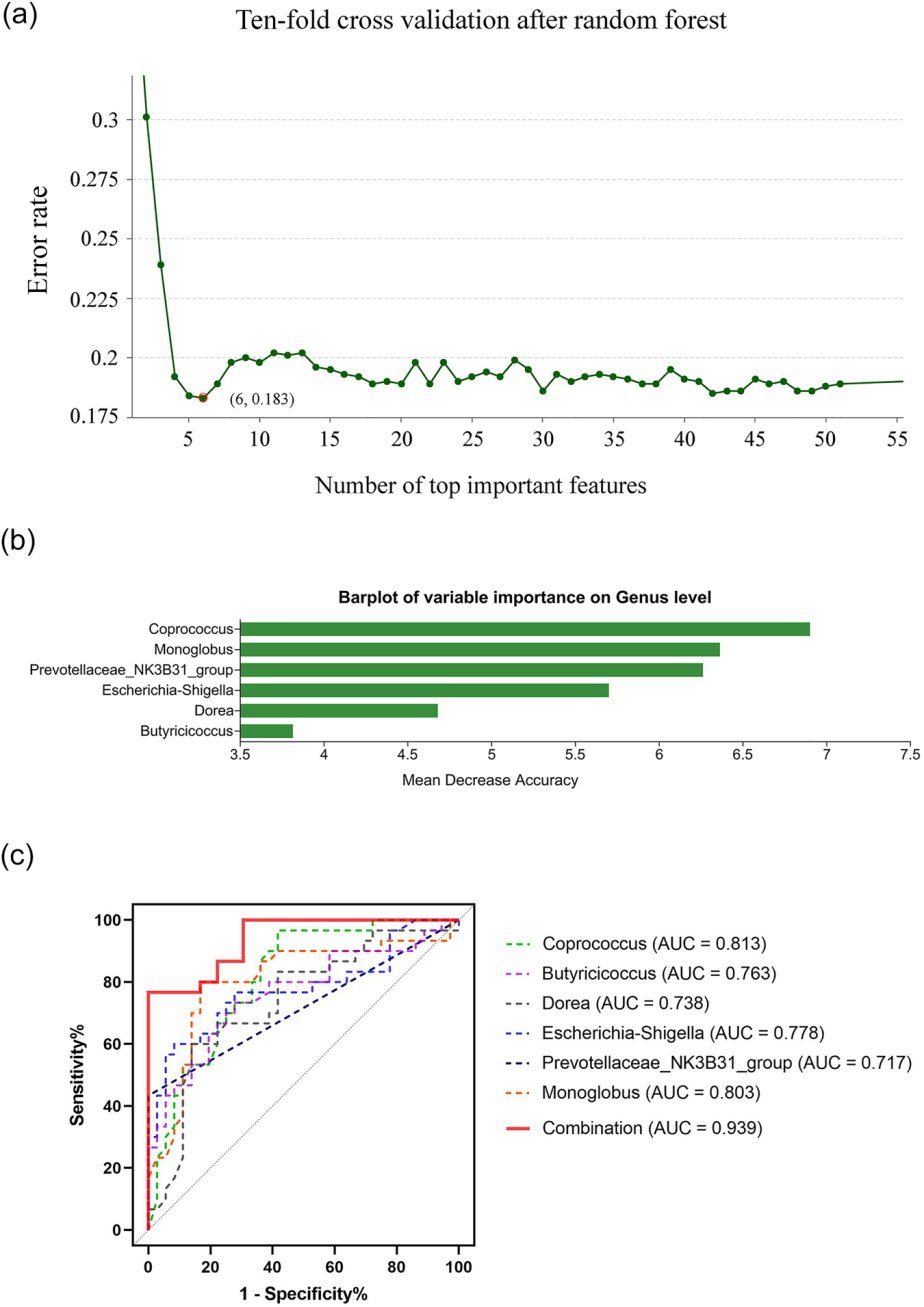
Potential gut microbial biomarkers of patients with schizophrenia with prominent negative symptoms (SCH‐N). (a) The six genera were selected as the optimal marker set by random forest models. (b) Species importance ranking chart. The *X*‐axis is the importance of the genus; the *Y*‐axis corresponds to the name of the genus in order of importance. (c) The AUC value reflects the diagnostic potential of microbial markers for discriminating SCH‐N from healthy control (HC) by receiver operating characteristic (ROC) analysis. The closer the AUC is to 1, the better the diagnosis. The AUC has some accuracy at 0.7 ∼ 0.9 and higher accuracy at AUC above 0.9.

After random forest analysis, six genera were selected by ten‐fold cross‐validation, which is shown in Figure 4a,b. Furthermore, we used the area under the ROC curve (AUC) to assess the diagnostic potential of microbial markers. *Coprococcus* (AUC = 0.813), *Monoglobus* (AUC = 0.803), *Prevotellaceae_NK3B31_group* (AUC = 0.717), *Escherichia‐Shigella* (AUC = 0.778), *Dorea* (AUC = 0.738), and *Butyricicoccus* (AUC = 0.763) could distinguish SCH‐N from HC. Finally, we found that the combined panel consisting of the above six genera showed a higher effectivity in distinguishing SCH‐N from HC (AUC = 0.939; 95% confidence interval: 0.886–0.991, Figure 4c).

In addition, we used random forest with ten‐fold cross‐validation to identify gut microbiota biomarkers that can distinguish SCH‐N and SCH‐P. We found that the minimum error rate was 0.544, indicating that these two groups cannot be effectively distinguished by the combination of gut microbiota in our study (Figure S1).

## 4 DISCUSSION

In this study, we investigated the microbial compositions and the associated clinical parameters in young adult SCH‐N. We found that the gut microbiomes could be separated clearly by PCoA. Moreover, there are differences between the two groups at the phylum, class, order, and family level. Finally, we identified 27 genera with different abundances and a specific microbial panel constituted of six genera, which may distinguish patients with SCH‐N from HCs with high reliability. These findings revealed that dysfunction of the gut microbiota might participate in the pathogenesis of SCH‐N, and the characteristic microbiota may be one of the potential peripheral markers to distinguish SCH‐N.

SCH was considered a neurodevelopmental disorder due to its high hereditability, which is estimated at around 80% (Owen & O'Donovan, 2017; Sullivan et al., 2003). Accumulated works also elucidate that several environmental factors, such as social isolation, substance abuse, or childhood trauma, play a role in how the disorder manifests (Chang et al., 2020; Stilo & Murray, 2019). Further research indicate that the imbalance of excitatory and inhibitory neurotransmission (Pafundo et al., 2021), oxidative stress (Ermakov et al., 2021), and neuroinflammation was involved in the pathogenesis of SCH (Vallee, 2022). Together, it is likely that SCH is a multifactorial disorder (Lahteenvuo & Tiihonen, 2021). Recently, the role of intestinal dysbacteriosis in SCH has been gradually becoming a concern. Besides the direct influence on energy metabolism, gut microbiota can also regulate oxidative stress, neuroinflammation, and neurotransmitter synthesis through its metabolites, such as short‐chain fatty acids (SCFAs) and kynurenine (Cryan et al., 2019; Jones et al., 2021; Tanaka et al., 2021). Consequently, intestinal dysbacteriosis was largely reported in patients with SCH (Samochowiec & Misiak, 2021), and transplantation of microbiota from drug‐free patients with SCH causes SCH‐like abnormal behaviors in mice (F. Zhu et al., 2020). Therefore, microbial characteristics are considered as potential biomarkers for diagnosing SCH (Shen et al., 2018).

SCH consists of symptoms from the positive, negative, and cognitive domains. Its primary pharmacological treatment is antipsychotics that work as dopamine D2‐receptor antagonists or partial agonists. However, these agents often could not markedly improve negative symptoms, suggesting that negative symptoms have different pathogenesis. Recent studies indicate that negative symptoms are likely to be the results of cortical glutamate deficiency (Coyle, 2012), hypodopaminergic function, cortical excitatory‐inhibitory imbalance (McCutcheon et al., 2020), and irregularities of distributed neural networks (e.g., cortico‐striatal, frontocortico‐temporal) (Millan et al., 2014). Moreover, a recent study compared the composition of the fecal microbiota of 82 patients with SCH (including 42 in the acute phase and 40 in remission) with 44 HCs and found that the abundance of *Haemophilus* was positively correlated, whereas *Coprococcus* was negatively correlated with negative symptoms (C. Zhu et al., 2021). Another study comparing differences in fecal microbiota between 25 chronic schizophrenic patients and 25 non‐psychotic subjects found that the *Ruminococcaceae* was negatively correlated with negative symptoms (Nguyen et al., 2019). However, the microbiota signatures of young adults with SCH‐N, as well as the potential microbial markers, still need to be elucidated.

To this end, the present study analyzed the composition difference of gut microbiota between SCH‐N patients and HCs, as well as between SCH‐N and SCH‐P in young adults. A previous study showed a higher α‐diversity in HCs than in SCH (R. Xu et al., 2020), and a recent work also suggested that low α‐diversity was associated with positive symptoms in SCH (Yuan et al., 2021). However, in the present study, there is no significant difference in the α‐diversity between HC and SCH‐N, and between SCH‐N and SCH‐P groups. Meanwhile, the clusters were separated clearly by β‐diversity, which is consistent with previous findings (Nikolova et al., 2021), suggesting α‐diversity is not a good discriminator of SCH‐N. The discrepant findings may be attributed to the differences in experimental design, characteristics of enrolled patients (age, gender, lifestyle, or first episode), and antipsychotic medication used. Regarding microbial taxa, we found a lower abundance of *Firmicutes* and a higher abundance of *Fusobacteria* and *Proteobacteria* phyla in SCH‐N, which was inconsistent with previous results investigated in patients with SCH (Shen et al., 2018). *Fusobacteria* contributes to the progression of cancer and has an impact on tumor immune response (Harrandah, 2023), and it is also enriched in patients with active major depressive disorder (MDD) (Jiang et al., 2015), whereas *Proteobacteria* contribute to airway inflammation and host metabolism (Cuesta et al., 2022; Dicker et al., 2021). It indicates that negative symptoms might also be related to inflammation.

In addition, we also discovered 27 genera that showed significant differences between HC and SCH‐N patients. *Escherichia‐Shigella*, *norank_f_Muribaculaceae*, and *Lachnoclostridium* were enriched in SCH‐N and were positively correlated with the total PANSS scores and negative subscales of PANSS scores. *Escherichia‐Shigella* is a hallmark of patients with IgA nephropathy and may serve as a promising diagnostic biomarker for IgA nephropathy (Zhao et al., 2022). It is already reported to be enriched in MDD and SCH (McGuinness et al., 2022). Meanwhile, *norank_f_Muribaculaceae* is related to chronic inflammatory bowel disease, whereas *Lachnoclostridium* is related to colitis, and basal levels of *Lachnoclostridium* are associated with the treatment response in SCH patients (L. Huang et al., 2022; Yuan et al., 2021). On the other hand, 10 of them were enriched in HCs and were negatively correlated with the total PANSS scores and negative subscales of PANSS scores (*Ruminococcus*, *Lachnospiraceae_NK4A136_group*, *Dorea*, *Butyricicoccus*, *Monoglobus*, *CAG‐352*, *Subdoligranulum*, *Ruminococcus_torques_group*, *Faecalibacterium*, and *Agathobacter*). Among them, *Ruminococcus* degrades polysaccharides and converts them into nutrients for hosts (La Reau & Suen, 2018). *Dorea* and *Faecalibacterium* are associated with glucose metabolism (Palmnas‐Bedard et al., 2022), and *Lachnospiraceae_NK4A136_group* is a SCFA‐producing bacterium (Ma et al., 2020). Together, SCH‐N might be closely related to intestinal health and metabolism. Notably, our study proposed a prediction model, which consisted of *Coprococcus*, *Monoglobus*, *Prevotellaceae_NK3B31_group*, *Escherichia‐Shigella*, *Dorea*, and *Butyricicoccus* and showed high accuracy (AUC = 0.939), indicating a powerful classification model. *Coprococcus* was associated with greater cardiovascular risk (Kelly et al., 2016) and was enriched in the HC group, and its abundance was negatively associated with negative psychiatric symptoms (C. Zhu et al., 2021). Meanwhile, it was consistently associated with higher quality of life indicators (Valles‐Colomer et al., 2019). *Dorea* and *Butyricicoccus* were related to butyrate production (Trachsel et al., 2018; Yi et al., 2021). The relative abundance of *Dorea* was increased after amisulpride treatment of acute exacerbated schizophrenic patients (J. Zheng et al., 2022), whereas *Butyricicoccus* was considered to be one of the SCH‐associated bacteria and enriched in HC (Ling et al., 2022). Intriguingly, the remaining two genera discovered in this study have not been reported previously in SCH. *Prevotellaceae_NK3B31_group* is a kind of butyric acid‐producing bacteria (P. Huang et al., 2021). A previous study found that its relative abundance was increased after bilateral common carotid artery occlusion, which was significantly reduced by fecal microbiota transplantation and replenishment of SCFAs (Su et al., 2022). Moreover, *Monoglobus* was positively associated with sleep efficiency (Magzal et al., 2022) and physical quality of life (Malan‐Muller et al., 2023). Therefore, negative symptoms might be related to the disturbance of SCFAs and sleep efficiency.

Furthermore, the difference in microbial composition between the SCH‐N and SCH‐P is much less than that between SCH‐N and the HC. Unfortunately, the present study found that SCH‐N and SCH‐P cannot be effectively distinguished by the combination of gut microbiota. This may be related to the inclusion criteria for patients in the SCH‐N and SCH‐P groups, such as the difference in PANSS scores between positive and negative patients in the two groups, which is only ≥3 points. Besides, negative symptoms are intrinsic to the underlying pathophysiology of SCH, and they also occur due to comorbidities and adverse effects of antipsychotics (Marder & Umbricht, 2023). Meanwhile, negative and positive symptoms are generally considered different manifestations of the same underlying condition rather than completely separate entities (Galderisi et al., 2018). Exploring special populations with obvious negative symptoms but few positive symptoms may be more reliable in explaining the relationship between negative symptoms and gut microbiota. Together, gut dysbiosis might be a characteristic of SCH in young adults, suggesting that strategies such as supplementing probiotics or SCFAs based on existing treatment methods may help alleviate negative symptoms.

Several shortcomings of the present study should be noted. This study is a cross‐sectional study, the course of illness varies greatly among patients, and most patients received medication treatment in the past. We cannot rule out the impact of previous antipsychotic medication on microbiota and negative symptoms (Y. Xu et al., 2022). Moreover, the impact of other factors, such as participants’ baseline diet and the usage of antibiotics and probiotics before fecal sample collection cannot be ruled out either (Leeming et al., 2019). It is necessary to study the characteristics of gut microbiota in first‐episode SCH patients through discovery and validation sets, which will help eliminate the interference of the aforementioned confounding factors. Meanwhile, the sample size appears relatively small for each group and each individual only collected fecal samples once without setting up replicates, which may contribute to overfitting in machine learning analysis like random forest classification and increase the randomness of the results and reduce the persuasiveness of this study. Finally, the 16S rRNA sequencing method may not be sensitive enough to detect small microbiota alterations (Ng, Lim, et al., 2023), and it also cannot accurately reflect the function of microbial communities. The combination of metagenomics with metabolomics (Ng, Yau, et al., 2023), such as lipidomics or proteomics, might further reveal the gut microbiota function and potential mechanisms of action in patients with SCH.

## 5 CONCLUSION

In summary, we characterized the composition of the gut microbiome in SCH‐N patients and developed a combined panel that could effectively distinguish SCH‐N from HC (consisting of *Coprococcus*, *Monoglobus*, *Prevotellaceae_NK3B31_group*, *Escherichia‐Shigella*, *Dorea*, and *Butyricicoccus*). Our results indicate that negative symptoms might be related to inflammation and the disturbance of SCFAs and sleep efficiency, which may shed light on the relationship between gut microbiota and SHC‐N. Novel intervention strategies that target microbiota might be promising in SCH‐N treatment, which needs to be further identified in the future.

## AUTHOR CONTRIBUTIONS

**Yi‐Huan Chen**: Conceptualization; investigation; writing—original draft. **Huan Yu**: Investigation; methodology. **Fen Xue**: Funding acquisition; validation. **Jie Bai**: Funding acquisition; validation; investigation. **Li Guo**: Conceptualization; investigation; writing—review and editing. **Zheng‐Wu Peng**: Conceptualization; investigation; supervision; funding acquisition; writing—original draft; writing—review and editing.

## CONFLICT OF INTEREST STATEMENT

The authors declare no conflicts of interest.

### Peer Review

The peer review history for this article is available at https://publons.com/publon/10.1002/brb3.3579.

## Supporting information

Supplementary Figure 1. Potential gut microbial biomarkers distinguishing SCH‐N and SCH‐P. (A) Only 1 genus was selected as the optimal marker set by random forest models. (B) Species importance ranking chart. The X‐axis is the importance of the genus.

Supplementary Table 1. Clinical and α‐diversity data

## Data Availability

The data that support the findings of this study are available from the corresponding author upon reasonable request.
