# Supplementary figures and images for: 16S rRNA gene sequencing reveals altered gut microbiota in young adults with schizophrenia and prominent negative symptoms
Source: Brain Behav. 2024 Jun 6;14(6):e3579. doi: 10.1002/brb3.3579 (PMC11154826; doi:10.1002/brb3.3579)

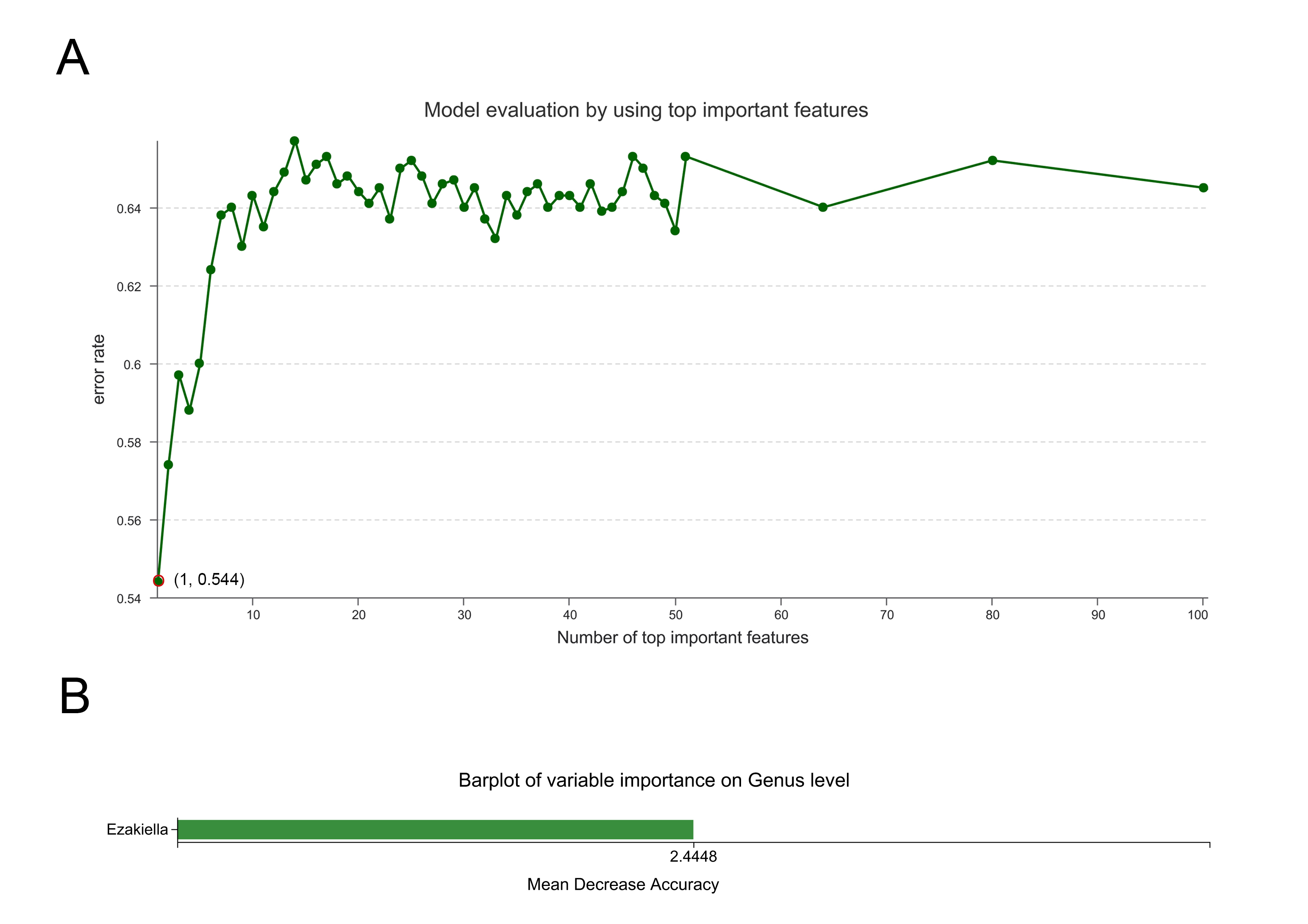

Supplement: Supplementary file 1 — Supplementary Figure 1. Potential gut microbial biomarkers distinguishing SCH‐N and SCH‐P. (A) Only 1 genus was selected as the optimal marker set by random forest models. (B) Species importance ranking chart. The X‐axis is the importance of the genus. [file BRB3-14-e3579-s002.jpg]
